# Amonabactin Synthetase G Regulates *Aeromonas hydrophila* Pathogenicity Through Modulation of Host Wnt/β-catenin Signaling

**DOI:** 10.3390/vaccines13020195

**Published:** 2025-02-17

**Authors:** Yiyang Tang, Xiaofeng Liu, Chuyi Zeng, Yujun Liu, Ye Yang, Jiayi Hu, Pingyuan Li, Zejun Zhou

**Affiliations:** 1 State Key Laboratory of Developmental Biology of Freshwater Fish, College of Life Sciences, Hunan Normal University, Changsha 410081, China; tyy0808@hunnu.edu.cn (Y.T.); chuyizeng@hunnu.edu.cn (C.Z.); yujunliu@hunnu.edu.cn (Y.L.); yangye0622@hunnu.edu.cn (Y.Y.); hujiayi0211@hunnu.edu.cn (J.H.); lipingyuan0902@hunnu.edu.cn (P.L.); 2 Department of Nutrition, National Clinical Research Center for Geriatric Disorders, Xiangya Hospital, Central South University, Changsha 410008, China; liuxiaofeng@csu.edu.cn; 3 Guangdong Provincial Key Laboratory of Aquatic Animal Disease Control and Healthy Culture, Guangdong Ocean University, Zhanjiang 524088, China

**Keywords:** siderophore, amonabactin, tight junction, gut, crucian carp

## Abstract

Background/Objectives: *Aeromonas hydrophila* is a significant opportunistic pathogen with a broad host range. It produces a catecholate siderophore, amonabactin, during iron starvation, but the in vivo infection mechanism that involves amonabactin is unclear. This study aims to elucidate the role of amonabactin synthetase G (AmoG) in the pathogenicity of *A. hydrophila* and its impact on gut barrier function. Methods: Δ*AmoG* was generated by deleting the AMP-binding domain of AmoG in *A. hydrophila* CCL1. In vivo infection experiments were conducted to assess the mutant’s iron-chelating ability and pathogenicity. Complementation of Δ*AmoG* with AmoG (Δ*AmoG*-C) was performed to confirm the observed phenotypes. Transcriptomic and qRT-PCR analyses were used to investigate gene expression changes in infected fish. Goblet cell counts, tight junction expression, and D-lactic acid and LPS levels were measured to evaluate gut barrier function. Results: Δ*AmoG* exhibited impaired iron-chelating ability and reduced pathogenicity compared to wild-type CCL1. Complementation with AmoG restored virulence in Δ*AmoG*-C. Transcriptomic and qRT-PCR analyses revealed an elevated expression of Wnt/β-catenin pathway components and antimicrobial genes in Δ*AmoG*-infected fish. Further investigation indicated increased goblet cells and an enhanced expression of tight junctions, as well as lower D-lactic acid and LPS levels, in Δ*AmoG*-infected fish. However, gut permeability, bacterial load, and lethality did not significantly differ between CCL1, Δ*AmoG*, and Δ*AmoG*-C infections when the Wnt/β-catenin pathway was activated. Conclusions: AmoG plays a crucial role in *A. hydrophila* pathogenicity by modulating host Wnt/β-catenin signaling and gut mucosal barrier function. This study provides insights into the pathogenesis of *A. hydrophila* and potential therapeutic targets.

## 1. Introduction

*Aeromonas hydrophila* is a ubiquitous pathogen within aquatic ecosystems and has led to substantial economic burdens for aquaculture [1,2,3]. Several prominent virulence factors have been identified, such as Type Three Secretion Systems (T3SSs) and T6SSs; toxins like Alt (heat-labile cytotonic enterotoxin), Act (cytotoxic enterotoxin), Ahp (serine protease); and various hemolysins [4]. In addition, siderophores are crucial for *A*. *hydrophila* to override the iron limitations imposed by the host or the environment [5]. Amonabactins, siderophores produced by the *Aeromonas* genus, can be used for iron acquisition to support a bacterium’s growth [6,7]. A previous study showed that *A*. *hydrophila* without the amonabactin biosynthesis gene *AmoG* was unable to synthesize any amonabactin or grow under iron stress conditions [8]. Nonetheless, the direct impact of *A. hydrophila*-produced amonabactin on host infection dynamics and its potential roles in modulating the fish epithelial barrier remain unexplored.

The integrity of the gut epithelial barrier is critical for preserving gut functionality and systemic immunity [9]. Pathogens employ different virulence mechanisms to disrupt epithelial junctions, thereby undermining gut barrier integrity [10,11,12]. *A. hydrophila*, notorious for its ability to severely compromise gut integrity and trigger septicemia, has been observed to cause gut lesions and induce gut inflammation in fish [13,14,15]. In a previous study, we identified a secretory serine protease (Ssp1) from *A. hydrophila* that plays a critical role in disrupting the gut’s tight junction barrier [16]. In addition, our earlier work pinpointed WbuB, a GT4 galactosaminogalactan synthase from *A. hydrophila*, as vital for inflammasome activation and gut tight junction disruption [17]. Nevertheless, the specifics of how *A. hydrophila* exploits nutrient acquisition, notably through amonabactin, to impact the gut mucosa barriers remain unclear.

In this study, we identified an amonabactin cluster with a potential iron uptake ability in *A. hydrophila* CCL1 (GenBank Accession No. CP092356). To elucidate this pathogenic mechanism, we selected the amonabactin biosynthesis gene *AmoG* for further study to determine whether it is a virulence factor in vivo. Through engineering Δ*AmoG* and its complement strain, comparative analyses encompassing physiological attributes and virulence capacities were conducted for these strains. Moreover, in vivo studies suggested that *A. hydrophila* may use amonabactin as one of its virulence factors to inhibit the Wnt/β-catenin pathway, consequently impairing the structure and function of the gut mucosal barrier. Our findings suggested that, unlike Ssp1 and WbuB, which predominantly influence the tight junction and inflammasome pathway, AmoG seems to manipulate the host Wnt/β-catenin signaling pathway through nutrient acquisition. These results provide key insights into gut mucosal dysfunction during *A. hydrophila* infection.

## 2. Materials and Methods

### 2.1. Fish

Crucian carps (~14.2 g) were obtained from Hunan Yuelu Mountain Science and Technology Co., Ltd.: Changsha, China, which carries out aquatic breeding in Changsha. During the experiment, fish were housed pre-experimentally under controlled conditions (25.25 ± 0.58 °C, pH 7.05 ± 0.12, >7.0 mg/L O_2_). Pre-experimental screening confirmed that the fish were disease-free [18]. Tissues were acquired following ethical guidelines (GB/T 35892-2018 [19]). All animal experiments were approved by the Hunan Normal University’s Animal Care Committee (No. 2023109).

### 2.2. Generation of A. hydrophila ΔAmoG and ΔAmoG-C

To generate the *A. hydrophila* Δ*AmoG* mutant, a 300 bp in-frame deletion of AmoG (aa 661–760) was engineered via an overlap extension PCR. The primers AmoG-F1/AmoG-R1 and AmoG-F2/AmoG-R2 were used sequentially for overlap PCRs, followed by a fusion PCR using AmoG-F1/AmoG-R2 (Table S1). The resultant amplicon was ligated into pDM4 at the BglII site, yielding pDMAmoG. *Escherichia coli* S17-1 λpir harboring pDMAmoG was conjugated with *A. hydrophila* CCL1. Following coculture and selection on polymyxin B/chloramphenicol plates, sucrose-resistant yet chloramphenicol-sensitive colonies were isolated. Confirmation of in-frame deletion in these isolates was achieved through PCR and sequencing, leading to the identification of the Δ*AmoG* mutant.

For the creation of the AmoG complement strain, designated Δ*AmoG*-C, the gene encoding AmoG, along with its native promoter, was PCR-amplified using the primers AmoG-F3 and AmoG-R3 (Table S1). This amplicon was then cloned into the pACYC184 plasmid at its EcoRV restriction site, forming pACYCAmoG-C. Subsequently, this construct was introduced into *E. coli* S17-1 λpir, which was subsequently mated with the Δ*AmoG* mutant. Transconjugants were identified on selective LB agar plates containing polymyxin B and chloramphenicol. One selected colony, designated Δ*AmoG*-C, was further validated by PCR and sequence confirmation.

### 2.3. Characterization of ΔAmoG and ΔAmoG-C

The strains CCL1, Δ*AmoG*, and Δ*AmoG*-C were cultured in LB broth at 28 °C for 24 h. Scanning electron microscopy was employed to assess cell morphology. Growth dynamics were monitored by cultivating the strains in LB with or without 100 μM 2, 2′-dipyridyl (Dp), starting at an A600 of 0.01; samples were taken at various intervals to measure A600. Motility assays and biofilm formation tests were conducted according to Reference [16]. The Chrome Azurol S (CAS) plate assay and Arnow’s test were used to analyze siderophore production according to Reference [20]. Each assay was replicated three times.

### 2.4. Cellular Adherence and Cytotoxicity Analyses

Caco-2 cells, obtained from American Type Culture Collection (ATCC, Manassas, VA, USA), were maintained in DMEM enriched with 20% FBS and antibiotics (penicillin/streptomycin) at 37 °C in 5% CO_2_. Approximately 1 × 10^5^ Caco-2 cells were seeded into 96-well plates and infected with CCL1, Δ*AmoG*, Δ*AmoG*-C, or PBS (control) at an MOI of 10:1. Cells were incubated for 1 h before washing away unbound bacteria using PBS. After infection, cells were lysed with Triton X-100, and the lysates were spread on LB agar to determine the bacterial counts. The adhesion index was calculated as the ratio of associated bacteria to Caco-2 cells. Each assay was replicated three times. Cytotoxicity was measured by the lactate dehydrogenase (LDH) method. Briefly, after 1 h of infection as above, LDH leakage was assessed using a Cytotoxicity Detection Kit (Roche Diagnostics, Mannheim, Germany). Cells treated with Triton X-100 represented the maximum LDH release (100%). Each assay was replicated three times.

### 2.5. In Vivo Infection

For in vivo infection, fish were injected intramuscularly (i.m.) with 100 µL suspensions containing 1 × 10^6^ CFU/mL of CCL1, Δ*AmoG*, Δ*AmoG*-C, or an equivalent volume of PBS (control). At 24 h post infection (hpi), distal gut, kidney, spleen, and blood from CCL1-, Δ*AmoG-*, Δ*AmoG*-C-, or PBS (control)-treated fish were carefully sampled under sterile conditions for subsequent experimental procedures. (i) Bacterial dissemination in the kidney and spleen and AB-PAS staining were carried out as reported previously [21]. (ii) A qRT-PCR was performed to analyze the genes’ expression in the distal gut. The genes and PCR primers used are listed in Table S1. Expression levels were measured through the 2^−ΔΔCT^ method, referencing beta-actin, with PCR efficiency (E) and correlation (R^2^) checked as previously detailed [22]. (iii) Plasma was derived from the blood of each group. D-lactic acid concentrations were measured using a D-lactic acid ELISA assay kit procured from Nanjing Jiancheng Bioengineering Institute, Nanjing, China. Additionally, Limulus Amebocyte Lysate (LAL) QCL-1000 kits from Lonza were employed to quantify lipopolysaccharide (LPS) levels within the plasma. (iv) An immunohistochemistry (IHC) assay was used to analyze the expression of Occludin in the distal gut as reported previously [16]. Mouse anti-rOccludin antibody (prepared in [16]) and Cy3-conjugated goat anti-mouse secondary antibody (Servicebio, Wuhan, China) were used as the primary and secondary antibodies, respectively. (v) Iron concentrations in the serum and distal gut were determined using an iron-unsaturated iron binding capacity kit (Nanjing Jiancheng) according to the manufacturer’s instruction. The DAB-enhanced Prussian blue iron staining kit (Servicebio, Wuhan, China) was utilized to detect iron deposits within liver samples according to the manufacturer’s instructions. (vi) To conduct an apoptosis assessment targeting the distal guts, an apoptosis assay was carried out using the TUNEL Apoptosis Detection Kit (Servicebio). (vii) For assessing survival rates, twenty crucian carps per group were challenged with CCL1, Δ*AmoG*, Δ*AmoG*-C, or received a PBS control, following the same inoculation procedure as described above. Over a period of two weeks, fish mortality was meticulously documented post infection as reported previously [21]. Each assay was replicated three times, with three fish used each time.

### 2.6. Gut Transcriptome and Microbiome Analyses

Transcriptome analysis was performed as outlined in previous work [16]. At 24 hpi, as above, distal guts from fish infected with CCL1, Δ*AmoG*, Δ*AmoG*-C, or exposed to PBS (serving as control) were collected under sterile conditions (with four fish in each group). Gut specimens from each group were frozen at −80 °C prior to RNA extraction for subsequent RNA sequencing (RNA-Seq). RNA extraction, library construction, and data analysis were performed by Novogene (Beijing, China).

For a comprehensive analysis of the microbiome shifts in the guts of fish exposed to CCL1, Δ*AmoG*, Δ*AmoG*-C, or treated with PBS (control), as above, distal guts from each group were taken and forwarded to Novogene for expert processing and sequencing (with four fish in each group). Following CTAB/SDS DNA extraction, libraries were constructed and sequenced using the 515F and 806R primer pair on an Illumina NovaSeq6000. Rigorous data analysis was performed through QIIME2 as reported previously [16].

### 2.7. Effect of the Activation of the Wnt/β-Catenin Pathway on Infection

Fish (twenty fish in each group) were injected i.m. with 100 µL of the Wnt/β-catenin agonist BIO (0.5 mg/mL, Sigma, St. Louis, MO, USA). Two hours following the initial procedure, each fish received an intramuscular injection comprising 100 µL of suspension, with concentrations of 1 × 10^6^ CFU/mL for either CCL1, Δ*AmoG*, Δ*AmoG*-C, or PBS. At 24 hpi, the kidney, spleen, and blood from CCL1-, Δ*AmoG-*, Δ*AmoG*-C-, or PBS (control)-injected fish were carefully sampled as above. Plasma LPS levels, bacterial dissemination, and survival rate assays were determined as reported above. Each assay was replicated three times.

### 2.8. Statistical Analysis

The sample size was calculated using PASS software (PASS 11. NCSS, LLC. Kaysville, UT, USA). Data were statistically analyzed using GraphPad Prism 9 (GraphPad Software, San Diego, CA, USA) and the Unpaired Mann–Whitney test or the Log-Rank test.

## 3. Results

### 3.1. Phenotypic Characterization of ΔAmoG

**Figure 1:**
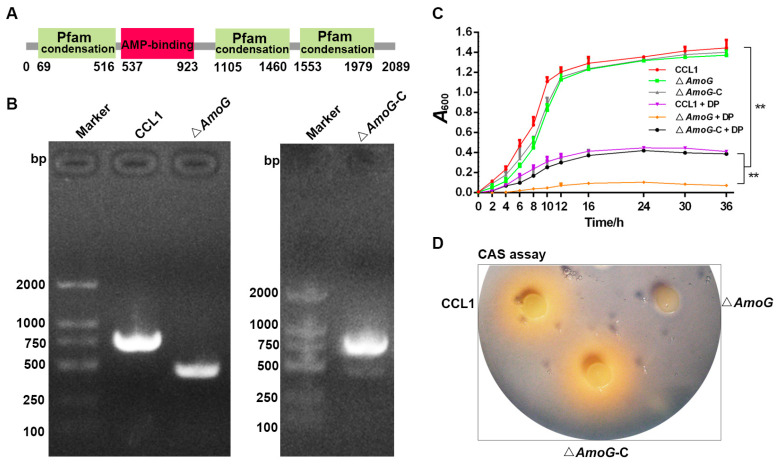
Generation of *Aeromonas hydrophila* Δ*AmoG* mutant and its complement strain Δ*AmoG*-C. (**A**) Depiction of AmoG’s domain. (**B**) PCR confirmation of the construction of Δ*AmoG* mutant and Δ*AmoG*-C by agarose gel electrophoresis analysis. (**C**) Growth levels of *A. hydrophila* CCL1, Δ*AmoG,* and Δ*AmoG*-C with or without 100 µM 2, 2′-dipyridyl (Dp). Values are Means ± SEM (N = 3). Mann–Whitney test. ** *p* < 0.01. (**D**) Representative photos of CCL1, Δ*AmoG,* and Δ*AmoG*-C and their siderophore production activity. CCL1, Δ*AmoG,* and Δ*AmoG*-C were spotted onto a filter disk in a CAS plate. One representative image from triplicate trials.

*A*. *hydrophila* produces a catecholate siderophore named amonabactin in response to iron starvation. A thorough examination of the genomic sequence of the fish pathogen *A*. *hydrophila* CCL1 (GenBank Accession No. CP092356) unveiled a gene cluster likely implicated in amonabactin production (Figure S1). This gene cluster possesses *AmoC*, *AmoE*, *AmoB*, *AmoF*, *AmoA*, *AmoG,* and *AmoH* (Figure S1). We focused on the potential amonabactin synthetase G (*AmoG*) from *A*. *hydrophila* CCL1 for further analysis. The deduced amino acid sequence of AmoG is composed of 2089 residues (GenBank Accession No. XIH98845.1). Sequence alignment shows that AmoG shares 94.5–26.4% overall sequence identities with homologs from *A*. *veronii*, *A*. *salmonicida*, *Vibrio parahaemolyticus*, *V*. *alginolyticus*, *V*. *harveyi*, *E. coli*, *Campylobacter jejuni,* and *Yersinia enterocolitica* (Figure S2). SMART could predict an AMP-binding domain (from 537 to 923 aa) and three Pfam condensations in AmoG (Figure 1A).

To probe AmoG’s role in pathogenicity, we engineered a variant of *A. hydrophila* CCL1, termed Δ*AmoG*, featuring an in-frame deletion of the AmoG segment (residues 661–760, Figure 1B). We also generated a complemented strain, Δ*AmoG*-C, reintroducing a functional AmoG to Δ*AmoG* (Figure 1B). Both Δ*AmoG* and Δ*AmoG*-C exhibited similar morphology and growth to their parental CCL1 in LB media (Table 1, Figure 1C), without noticeable changes in biofilm formation or swimming motility (Table 1). While CCL1 had an LD_50_ of 9.26 × 10^4^ CFU/fish, Δ*AmoG* displayed a reduced virulence, with an LD_50_ of 8.58 × 10^5^ CFU/fish, indicating a roughly ten-fold decrease (Table 1). In addition, under the iron-depleted condition imposed by adding 100 µM Dp, both CCL1 and Δ*AmoG*-C demonstrated a suppressed growth level; however, Δ*AmoG* was virtually incapable of growing in such an environment (Figure 1C). Furthermore, Δ*AmoG* failed to produce the expected yellow hue in CAS medium, indicating its role in iron–siderophore interactions (Figure 1D). This observation underscores AmoG’s critical role in facilitating metabolic adaptations for iron uptake, revealing its importance in enabling survival under conditions of iron scarcity.

### 3.2. AmoG-Mediated Iron Chelation and Bacterial Infection

**Figure 2:**
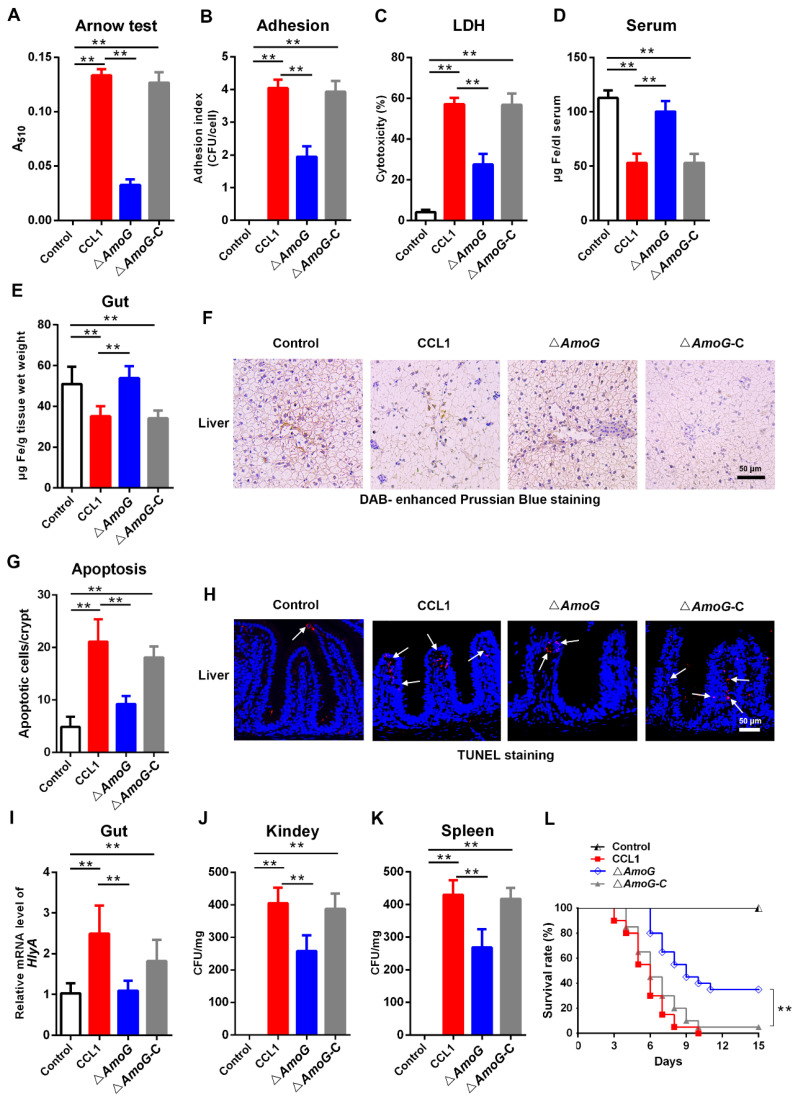
Effect of AmoG on bacterial infection. (**A**) An Arnow assay was used to analyze the production of amonabactin in *Aeromonas hydrophila* CCL1, Δ*AmoG,* and Δ*AmoG*-C. Caco-2 cells were challenged with CCL1, Δ*AmoG*, Δ*AmoG*-C, and PBS (control) for 1 h, and their (**B**) adhesion index and (**C**) cytotoxicity were determined. Crucian carps were challenged with CCL1, Δ*AmoG*, Δ*AmoG*-C, or PBS (control) for 24 h. Iron contents in the (**D**) serum, (**E**) distal gut, and (**F**) liver were analyzed. (**G**,**H**) The apoptotic cells in the distal guts of infected fish were examined using a TUNEL analysis. White arrow indicates apoptotic cells. The bacterial burdens in the distal guts (**I**), kidneys (**J**), and spleens (**K**) were measured. Values are Means ± SEM (N = 3). Mann–Whitney test. ** *p* < 0.01. (**L**) Survival curves. Fish infected with CCL1, Δ*AmoG*, Δ*AmoG*-C, and PBS (control) were monitored over two weeks. Log-rank test (*p* < 0.01).

Following the above findings, which suggested a deficiency in the iron-chelating process of the Δ*AmoG* strain, we proceeded to investigate whether there was an alteration in its capability to produce siderophore. The Arnow assay confirmed that Δ*AmoG* exhibited a significantly diminished ability to synthesize the iron-chelating siderophore amonabactin (Figure 2A). When incubated with Caco-2 cells, an intestinal epithelial cell line, notably reduced adherence and cytotoxicity were found in the ∆*AmoG*-treated group compared to the CCL1- and Δ*AmoG*-C-treated groups (Figure 2B,C). Moreover, iron levels in the serum, gut, and liver of fish infected with CCL1 and ∆*AmoG*-C were substantially lower than those seen after infection with ∆*AmoG* (Figure 2D–F). This finding suggested that the deletion of AmoG in *A. hydrophila* led to an accumulation of iron in these tissues, potentially due to the impaired iron-chelating abilities of the bacteria. Following up on these observations of the effects of AmoG on the iron-chelating process, a TUNEL analysis was used to investigate cell apoptosis under iron deficiency conditions. The result showed that distal gut apoptosis was significantly lower in ∆*AmoG*-infected fish compared to those with CCL1 or ∆*AmoG*-C infections (Figure 2G,H). Upon analyzing the bacterial loads within the distal guts, kidneys, and spleens of infected fish, we observed considerably lower numbers of *A. hydrophila* in those infected with the ∆*AmoG* strain (Figure 2I–K). Correspondingly, there was a notable decline in mortality among ∆*AmoG*-infected fish, indicating that the virulence of ∆*AmoG* had been significantly dampened (Figure 2L). These results suggest that *A. hydrophila* may employ AmoG in the synthesis of amonabactin to facilitate iron chelation, consequently impairing the structure and function of the gut mucosal barrier.

### 3.3. Transcriptomic Analysis

**Figure 3:**
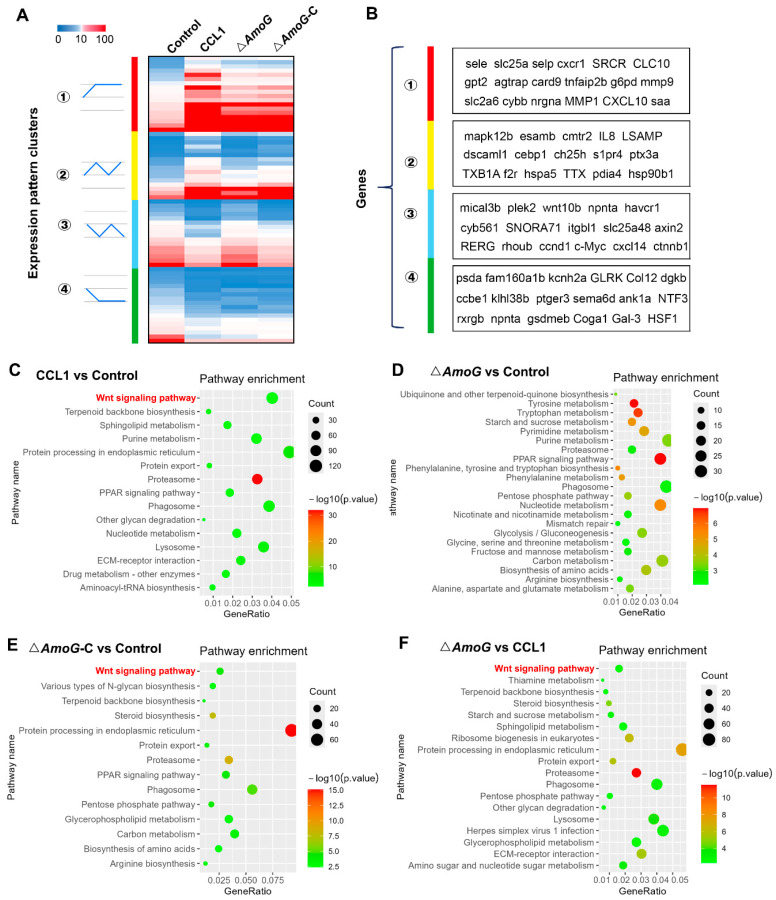
Transcriptomic impact of AmoG on the distal gut during *Aeromonas hydrophila* infection. Crucian carps were challenged with *A. hydrophila* CCL1, Δ*AmoG*, Δ*AmoG*-C, or PBS (control) for 24 h, and a transcriptomic analysis of their distal gut was performed. (**A**) Heatmap representations of differentially expressed genes upon infection with CCL1, Δ*AmoG*, Δ*AmoG*-C, or PBS. Clustering reveals the genes sharing the same expression trends. Mann–Whitney test, *p* < 0.01. (**B**) Highlighted genes exhibiting notable expression changes within identified clusters. (**C**–**F**) KEGG pathway enrichment analysis identifying the Wnt signaling pathway as being significantly affected during *A. hydrophila* infection.

To further investigate the pathogenic mechanism orchestrated by AmoG, we conducted transcriptomic analyses on the distal guts of *A. hydrophila*-infected crucian carps. The results revealed that compared to Δ*AmoG* infection, CCL1 and Δ*AmoG*-C infection notably repressed the expression of several key genes, including *wnt10b*, *axin2*, *ccnd1,* and *ctnnb1*, whereas genes like *IL-8*, *ptx3a,* and *hsp90b1* experienced pronounced upregulation (Figure 3A,B). A further KEGG pathway analysis highlighted that the Wnt/β-catenin signaling pathway (a key regulator of cell proliferation, differentiation, and homeostasis) was significantly impacted during Δ*AmoG* infection, underscoring its pivotal role in mediating disease progression and the host’s response (Figure 3C–F). This finding also suggested a possible connection between the AmoG-mediated iron-chelating process and the Wnt/β-catenin signaling pathway.

### 3.4. Impact of AmoG on the Wnt/β-Catenin Pathway

**Figure 4:**
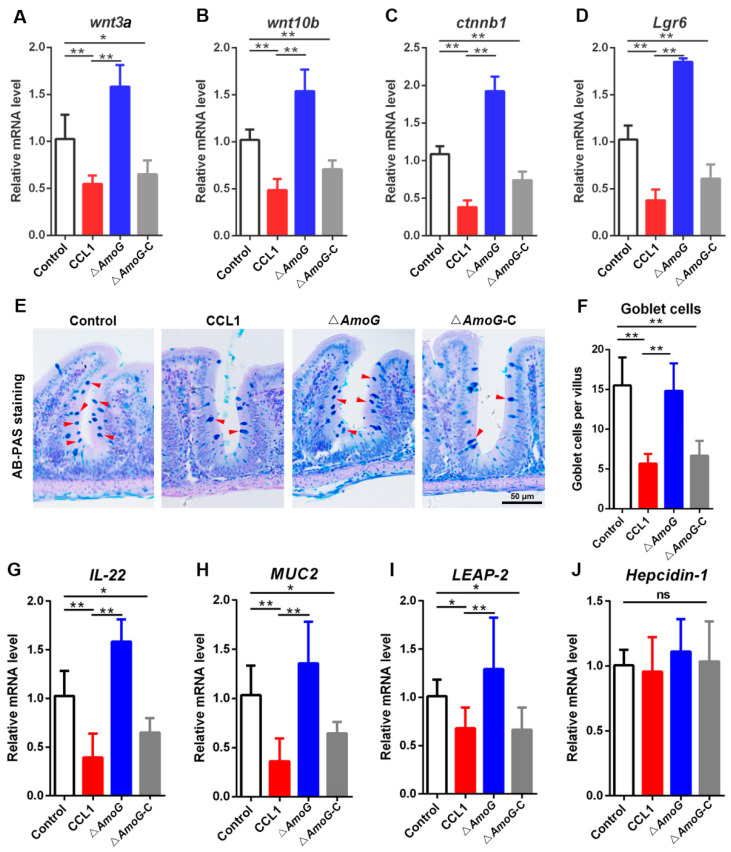
Effect of AmoG on goblet cells and antimicrobial molecules in the distal gut. Crucian carps were challenged with *Aeromonas hydrophila* CCL1, Δ*AmoG*, Δ*AmoG*-C, or PBS (control) for 24 h. (**A**–**D**) The expression of *wnt3a*, *wnt10b*, *ctnnb1*, and *Lgr6* in the distal guts was analyzed by qRT-PCR. (**E**,**F**) AB-PAS staining was used to show the goblet cells in the distal guts. Red arrow indicates goblet cells. Bar: 50 μm. (**G**–**J**) The expression of antimicrobial genes in the distal guts was analyzed by qRT-PCR. Values are Means ± SEM (N = 3). Mann–Whitney test. * *p* < 0.05; ** *p* < 0.01; ns: no significance.

The Wnt/β-catenin signaling pathway is fundamental for the regeneration of the gut mucosal barrier. Given that transcriptomic data suggested a disruption of the Wnt/β-catenin pathway during *A. hydrophila* infection, we proceeded to examine the effect of the Wnt/β-catenin pathway on the gut mucosal barrier. Initially, consistent with the transcriptomic data, key components of the Wnt/β-catenin pathway, such as *wnt3a*, *wnt10b*, *ctnnb1*, and *Lgr6*, displayed a significantly upregulated expression in the fish treated with Δ*AmoG* relative to those treated with CCL1 and Δ*AmoG*-C (Figure 4A–D). Subsequently, AB-PAS staining indicated that the counts of distal gut goblet cells in Δ*AmoG*-infected fish notably surpassed those seen in CCL1- and Δ*AmoG*-C-infected fish (Figure 4E,F). IL-22, known to foster gut epithelial cell proliferation and antimicrobial peptide synthesis [23], also exhibited heightened expression in the distal guts following Δ*AmoG* administration (Figure 4G). Moreover, a qRT-PCR revealed an upsurged expression of *MUC2*, *LEAP-2*, and *Hepcidin-1* in Δ*AmoG*-treated fish compared to CCL1- or Δ*AmoG*-C-treated fish (Figure 4H–J). Conversely, fish treated with CCL1 or Δ*AmoG*-C demonstrated a suppressed expression of these genes. These results indicated that without AmoG as a key virulence factor to synthesize amonabactin, the ∆*AmoG* mutant’s capacity to inhibit the Wnt/β-catenin pathway was markedly reduced.

### 3.5. Impact of AmoG on Gut Mucosal Barrier

**Figure 5:**
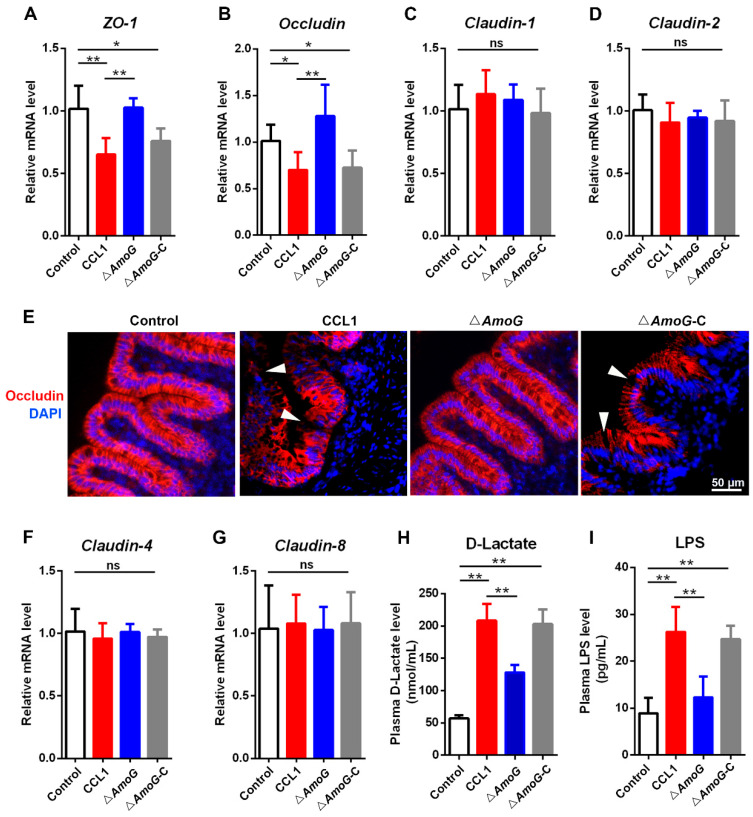
Effect of AmoG on the gut mucosa barrier. Crucian carps were challenged with *Aeromonas hydrophila* CCL1, Δ*AmoG*, Δ*AmoG*-C, or PBS (control) for 24 h. The expression of (**A**) *ZO-1*, (**B**) *Occludin*, (**C**) *Claudin-1*, (**D**) *Claudin-2*, (**F**) *Claudin-4,* and (**G**) *Claudin-8* in the distal guts was determined by qRT-PCR. (**E**) Immunohistochemistry demonstrates Occludin’s distribution in the distal guts. White arrow indicates the location of Occludin. Gut permeability was measured by (**H**) plasma D-lactic acid and (**I**) LPS levels. Values are Means ± SEM (N = 3). Mann–Whitney test. * *p* < 0.05, ** *p* < 0.01. ns: no significance.

Given the marked reduction in Δ*AmoG*’s capacity to inhibit the Wnt/β-catenin pathway, we proceeded to investigate its influence on the gut mucosal barrier. THe qRT-PCR showed that a heightened expression of *Occludin* was observed in the distal gut of Δ*AmoG*-exposed fish, compared to that in both the CCL1 and Δ*AmoG*-C groups (Figure 5A,B). The IHC analysis also demonstrated that, unlike in CCL1- or Δ*AmoG*-C-treated fish, Occludin, a vital tight junction constituent, was markedly upregulated in the distal guts of Δ*AmoG*-treated fish (Figure 5E). Additionally, plasma D-lactic acid and LPS, markers of gut lining integrity [24], were notably lower in Δ*AmoG*-infected fish compared to those with CCL1 and Δ*AmoG*-C infections (Figure 5H,I). Since disrupted gut barrier function can lead to alterations in the gut microbiota, we also investigated the gut microbiome differences among fish infected with CCL1, Δ*AmoG*, or Δ*AmoG*-C (Figure S3). The results showed that CCL1 infection led to discernible alterations in the gut microbiota compared to Δ*AmoG* infection, indicating that the ∆*AmoG* mutant exhibited a diminished ability to compromise the gut mucosal barrier.

### 3.6. Impact of the Wnt/β-Catenin Pathway on Bacterial Infection

**Figure 6:**
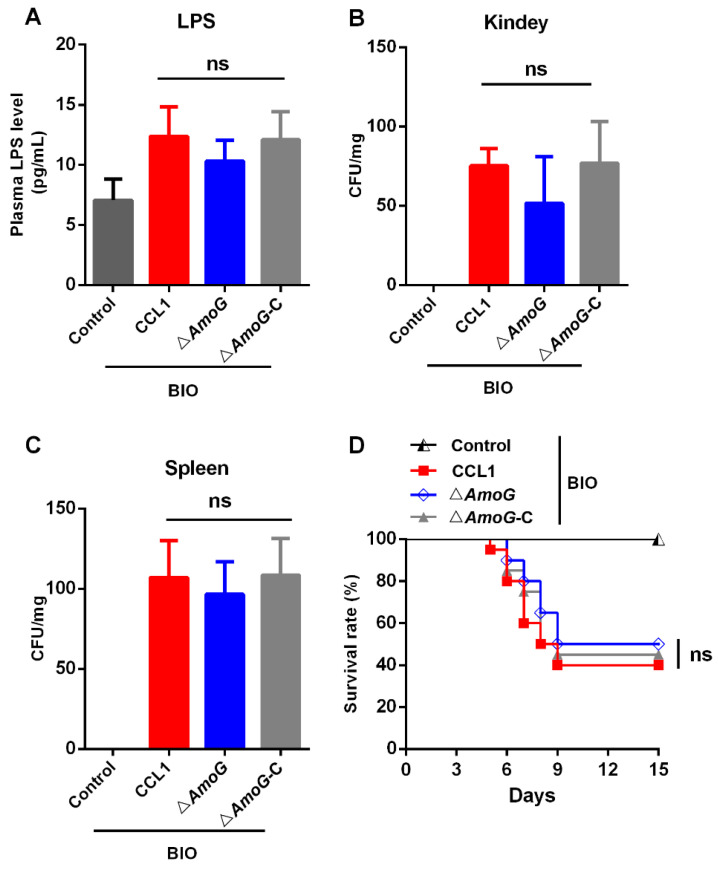
Effect of the Wnt signaling pathway on antibacterial immunity. Crucian carps were pre-treated with the Wnt/β-catenin pathway activator BIO (0.5 mg/mL) and were subsequently challenged with *Aeromonas hydrophila* CCL1, Δ*AmoG*, Δ*AmoG*-C, or PBS (control) for 24 h. (**A**) Gut permeability was analyzed using plasma LPS levels. The bacterial burdens in the (**B**) kidneys and (**C**) spleens were determined. Values are Means ± SEM (N = 3). Mann–Whitney test, ns: no significance. (**D**) Survival curves. Crucian carps were pre-treated with the Wnt/β-catenin pathway activator BIO as above and then were challenged with CCL1, Δ*AmoG*, Δ*AmoG*-C, or PBS (control). Fish were monitored over two weeks. Log-rank test (*p* < 0.01), ns: no significance.

To further explore the impact of Wnt/β-catenin signaling pathway activation on *A. hydrophila* infection, we pre-treated fish with the Wnt/β-catenin pathway activator BIO before infecting them with either CCL1, Δ*AmoG*, or Δ*AmoG*-C. Our results revealed that upon activation of the Wnt/β-catenin signaling pathway, the gut permeability of these fish, as well as the bacterial burdens in their kidneys and spleens, showed no significant differences among the infections caused by CCL1, Δ*AmoG*, or Δ*AmoG*-C (Figure 6A–C). Similarly, the lethal effects attributed to each bacterial strain remained statistically indistinguishable following the activation of the Wnt/β-catenin pathway (Figure 6D). In summary, these findings suggest that once the Wnt/β-catenin pathway is activated, the presence or absence of amonabactin does not appear to significantly influence the bacterium’s infectivity. Additionally, these results suggest that *A. hydrophila* may employ amonabactin as a strategic tool to dampen the Wnt/β-catenin signaling pathway, which in turn facilitates the bacterium’s ability to be invasive, making this a probable mechanism underlying its pathogenicity (Figure S4).

## 4. Discussion

Iron is an essential trace element for most organisms [25,26,27]. In response to iron starvation, *A*. *hydrophila* has been shown to possess multiple systems for the sequestration of host iron, including heme-bound iron transport [28], the utilization of enterobactin siderophores produced by Enterobacteriaceae [6,7], and the secretion of amonabactin [29]. Among these virulence factors, amonabactin represents a family of catechol peptidic siderophores that is composed of seven genes named *AmoCEBFAGH* [8]. A previous study indicated that a mutant of AmoG was unable to synthesize any amonabactin or to grow in iron-stress conditions [8]. Consistent with this study, our results showed that under a treatment with 100 µM Dp, Δ*AmoG* was almost unable to grow, indicating that the AmoG of *A*. *hydrophila* CCL1 may be involved in bacterial iron acquisition and survival under iron stress.

Until now, AmoG’s in vivo function has remained unexplored experimentally. To explore this, firstly, we embarked on a comprehensive transcriptomic analysis targeting the guts of crucian carps infected with *A. hydrophila*. Our findings unveiled that in comparison to Δ*AmoG* infections, CCL1 and Δ*AmoG*-C infections markedly suppressed the expression of pivotal genes in the Wnt/β-catenin signaling pathway including *wnt10b*, *axin2*, *ccnd1,* and *ctnnb1*. The Wnt/β-catenin pathway is integral to the structural integrity of the gut barrier and is essential for the proper functioning of gut epithelial cells, such as goblet cells [30]. In line with the above observations, we observed a significant decline in goblet cells and *MUC2*, *IL-22*, *Hepcidin-1,* and *LEAP-2* expression in fish subjected to CCL1 or Δ*AmoG*-C infection. These results indicated that AmoG may be involved in the pathogenicity of *A*. *hydrophila* infection through the inhibition of the Wnt/β-catenin pathway. Secondly, the Wnt/β-catenin pathway promotes the assembly and stability of gut tight junctions, ensuring minimal paracellular leakage [31,32]. Previous investigations have documented the capacity of specific pathogens, notably *Helicobacter pylori* and *Campylobacter jejuni*, to inhibit the Wnt/β-catenin pathway and compromise the integrity of tight junctions during the course of their infection [33]. For this purpose, we analyzed the impact of AmoG on gut permeability in an in vivo setting. Our findings revealed a significant upregulation of occludin in fish infected with Δ*AmoG*. Consistently, the decreased concentrations of plasma D-lactic acid and LPS indicated a reduced gut mucosal permeability following Δ*AmoG* infection. Moreover, our data demonstrated a slight but notable enhancement in alpha diversity within the gut flora of fish infected with Δ*AmoG* relative to those harboring CCL1, hinting at potential disturbances in bacterial colonization elicited by AmoG [34,35]. Collectively, the above results suggested that AmoG might be involved in the infection process, particularly under conditions of an inhibited Wnt/β-catenin pathway and disrupted tight junction barriers. However, additional studies are warranted to validate whether AmoG’s effects are direct or mediated indirectly through other pathways.

Iron is an indispensable micronutrient for bacterial metabolism, proliferation, and infection. Bacterial pathogens have developed intricate systems for iron acquisition, employing iron carriers known as siderophores to chelate and sequester iron from the host environment, influencing their infection capacities [36,37]. Similarly, our results demonstrated that Δ*AmoG* infection leads to elevated iron levels in the serum, gut, and liver of infected fish, possibly due to its impaired iron-chelating efficiency, which may subsequently affect the infection dynamics of *A. hydrophila*. Indeed, upon infecting fish with Δ*AmoG*, we observed notably smaller bacterial counts in their tissues compared to those in fish infected with *CCL1*. The reason may be that the iron-chelating process used by *A. hydrophila* enables the bacterium to disrupt the gut barrier and simultaneously enhance its invasive capabilities. Research suggests that the inhibition of the Wnt/β-catenin signaling pathway by pathogens through iron chelation provides the bacteria with an advantage in disrupting the gut barrier, thereby enhancing their invasiveness [38]. Studies have reported that a shortage of iron, brought about by siderophores, can lead to the suppression of the Wnt/β-catenin signaling pathway, favoring bacterial infection [39,40]. Consistent with these findings, our findings revealed that once the Wnt/β-catenin pathway is activated, the presence or absence of amonabactin does not seem to significantly alter the bacterium’s infectivity. This observation further points to the possibility that *A. hydrophila* may use amonabactin as one of its virulence factors to inhibit the Wnt/β-catenin pathway and consequently impair the structure and function of the gut mucosal barrier. In addition, a study on *Edwardsiella piscicida* highlights the role of Fur in iron regulation and virulence, similar to the role of AmoG in *A. hydrophila*. In the future, using siderophore-deficient strains as vaccines may be an effective prevention and control strategy in aquaculture [41].

Interestingly, our previous studies have identified Ssp1 and WbuB as essential components for disrupting the gut’s tight junction barrier [16,17]. In contrast to Ssp1 and WbuB, which primarily influence the tight junction and inflammasome pathway, AmoG uniquely targets the Wnt/β-catenin signaling pathway. Through iron acquisition, AmoG appears to manipulate the host’s Wnt/β-catenin signaling pathway, thereby facilitating both pathogenesis and nutrient acquisition. Further exploration is needed to understand the precise mechanisms linking iron chelation to the mediation of the Wnt/β-catenin signaling pathway.

## 5. Conclusions

In conclusion, our findings indicate that AmoG is a previously unrecognized determinant in the pathogenicity of *A. hydrophila*, essential for successful host invasion. Our observations highlight AmoG’s substantial disruption of the Wnt/β-catenin signaling pathway and gut barrier integrity. Moreover, the diminished expression of AmoG correlates with reduced *A. hydrophila* colonization in key immune tissues, significantly attenuating its virulence. These discoveries offer new perspectives on *A. hydrophila*’s pathogenesis, contributing valuable knowledge to the prevention and treatment of this aquatic pathogen.

## Figures and Tables

**Table 1:** Characteristics of the *Aeromonas hydrophila AmoG* mutant.

Characteristics	CCL1	Δ*AmoG*	Δ*AmoG*-C
Morphology ^a^	Ns	Ns	Ns
Biofilm formation ^b^	0.25 ± 0.09	0.25 ± 0.05	0.25 ± 0.05
Swimming motility (mm) ^c^	13.1 ± 0.15	12.8 ± 0.22	12.9 ± 0.19
LD_50_ (CFU/fish) ^d^	9.26 × 10^4^	8.58 × 10^5^ **	1.08 × 10^5^

Note: Values are Means ± SD of three trials. Mann–Whitney test or log-rank test. Ns: no significance. ** *p* < 0.01. ^a^: Cell morphology was examined using electronic scanning microscopy after culturing in LB medium for 24 h at 28 °C. ^b^: Bacteria were incubated in 96-well polypropylene plates for 24 h at 28 °C. ^c^: The diameters of swarming colonies were measured after 24 h of incubation on LB plates containing 0.3 % agar. ^d^: The LD₅₀ values were determined in healthy crucian carp weighing an average of 14.2 g.

## Data Availability

The raw data from the RNA-Seq and 16S rDNA Amplicon Sequencing have been uploaded to the NCBI Sequence Read Archive under accession numbers PRJNA1192974 and PRJNA807750.

## Supplementary Materials

The following supporting information can be downloaded at: https://www.mdpi.com/article/10.3390/vaccines13020195/s1, Figure S1: Genomic organization of the amonabactin synthesis gene cluster in *Aeromonas hydrophila* CCL1 (GenBank Accession No. CP092356); Figure S2: Alignment of AmoG; Figure S3: Effect of AmoG on gut microbiota in vivo; Figure S4: Summary depiction suggesting a possible mechanism by which *Aeromonas hydrophila* AmoG modulates the infectivity through the manipulation of the Wnt/β-catenin pathway; Table S1: PCR primers used in this study.
