# Supplementary material for: Amonabactin Synthetase G Regulates Aeromonas hydrophila Pathogenicity Through Modulation of Host Wnt/β-catenin Signaling
Source: Vaccines (Basel). 2025 Feb 17;13(2):195. doi: 10.3390/vaccines13020195 (PMC11861348; doi:10.3390/vaccines13020195)
Supplement: Supplementary file 1 [file vaccines-13-00195-s001.zip › Figure S1.pdf]

Figure S1. Genomic organization of the amonabactin synthesis gene cluster in *Aeromonas hydrophila* CCL1 (GenBank Accession No. CP092356). The arrows represent the genes and their orientations.

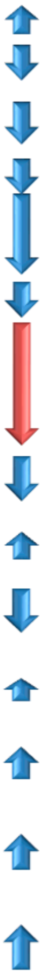

Map	Locus	GenBank annotation	SwissProt
YceB	Chr1:2111332:2111883:-	WP_016350965.1; hypothetical protein	Uncharacterized lipoprotein YceB
AmoC	Chr1:2112224:2113402:+	WP_043119102.1; isochorismate synthase	Amonabactin synthetase C, AmoC
AmoE	Chr1:2113399:2115060:+	WP_016350963.1; 2, 3-dihydroxybenzoate-AMP ligase	Amonabactin synthetase E, AmoE
AmoB	Chr1:2115084:2116007:+	WP_016350962.1; isochorismatase	Amonabactin synthetase B, AmoB
AmoF	Chr1:2116004:2119093:+	WP_016350961.1; non-ribosomal peptide synthetase	Amonabactin synthetase F, AmoF
AmoA	Chr1:2119114:2119863:+	WP_016350960.1; 2, 3-dihydro-2, 3-dihydroxybenzoate dehydrogenase	Amonabactin synthetase A, AmoA
AmoG	Chr1:2119878:2126147:+	WP_016350959.1; non-ribosomal peptide synthetase	Amonabactin synthetase G, AmoG
AmoH	Chr1:2126144:2127688:+	WP_016350958.1; dimodular nonribosomal peptide synthetase	Amonabactin synthetase H, AmoH
FecB	Chr1:2127764:2128708:-	WP_016350957.1; ABC transporter substrate-binding protein	Amonabactin ABC transporter substrate-binding protein
AmoD	Chr1:2128868:2129650:+	WP_016350956.1; 4'-phosphopantetheinyl transferase	Amonabactin transferase, AmoD
YusV	Chr1:2129712:2130515:-	WP_016350955.1; iron-siderophore ABC transporter ATP-binding protein	Amonabactin ABC transport system ATP-binding protein YusV
FepG	Chr1:2130559:2131626:-	WP_016350954.1; MULTISPECIES: iron ABC transporter permease	Amonabactin ABC transporter permease subunit 1
FepD	Chr1:2131640:2132620:-	WP_011705838.1; ABC transporter, iron chelate uptake transporter (FeCT) family, permease protein	Amonabactin ABC transporter permease subunit 2
CirA	Chr1:2132725:2134698:-	WP_016350953.1; TonB-dependent siderophore receptor	Amonabactin TonB-dependent receptor
