# Supplementary material for: Amonabactin Synthetase G Regulates Aeromonas hydrophila Pathogenicity Through Modulation of Host Wnt/β-catenin Signaling
Source: Vaccines (Basel). 2025 Feb 17;13(2):195. doi: 10.3390/vaccines13020195 (PMC11861348; doi:10.3390/vaccines13020195)

Figure S2. Alignment of AmoG. Red box represents the AMP-binding domain. Identities of AmoG between *Aeromonas hydrophila* and compared species are placed at the end of each species. The GenBank accession numbers are as follows: *A. hydrophila*, XIH98845.1; *A. veronii*, MFM5620825.1; *A. salmonicida*, MCE9969620.1; *Vibrio parahaemolyticus*, EOG7722708.1; *V. alginolyticus*, WP_390509635.1; *V. harveyi*, WP_017189278.1; *Escherichia coli*, EGK4048697.1; *Campylobacter jejuni*, EIJ4040616.1; *Yersinia enterocolitica*, HDL7775937.1.

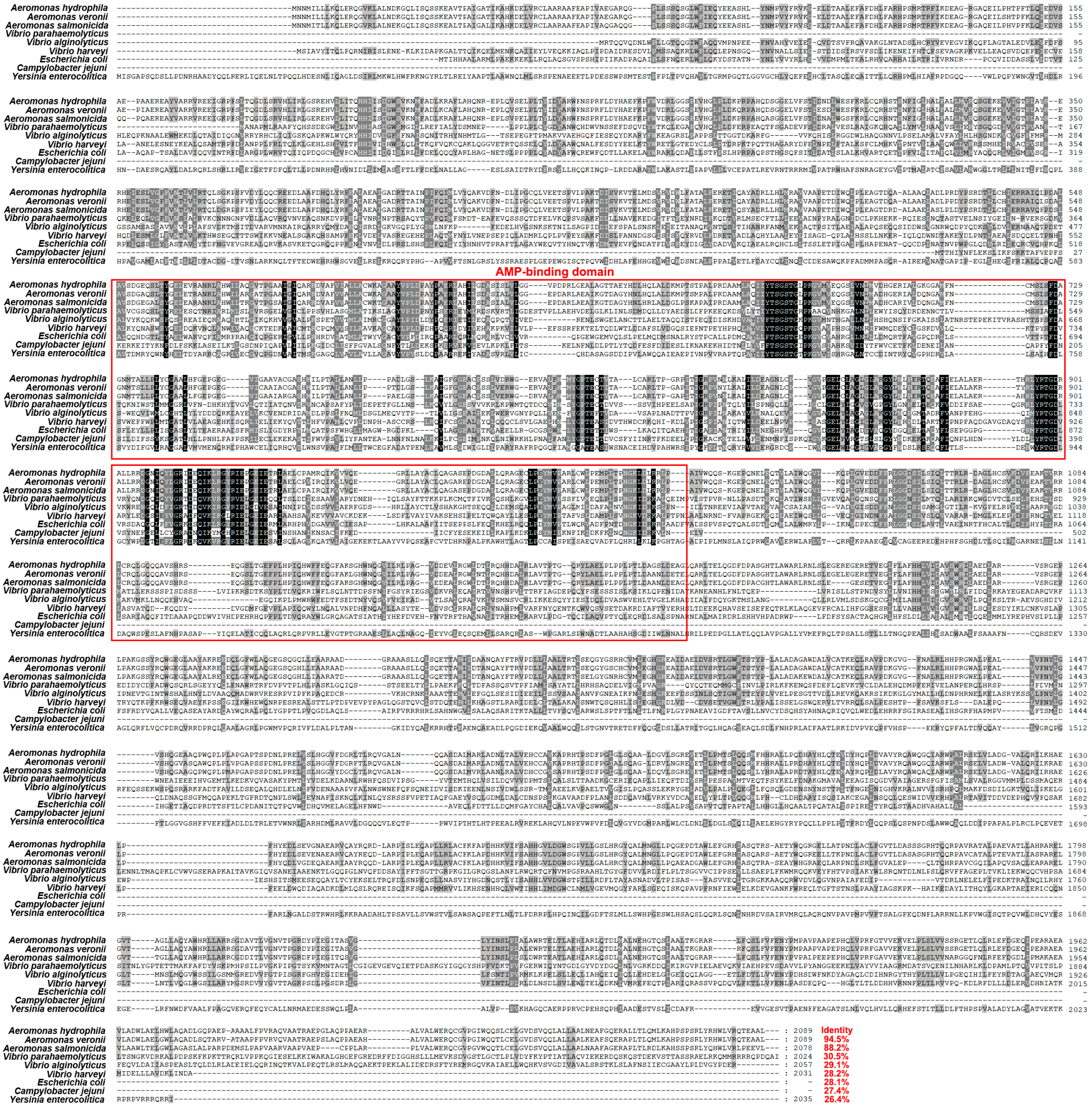

Supplement: Supplementary file 1 [file vaccines-13-00195-s001.zip › Figure S2.pdf]
