# Supplementary material for: Amonabactin Synthetase G Regulates Aeromonas hydrophila Pathogenicity Through Modulation of Host Wnt/β-catenin Signaling
Source: Vaccines (Basel). 2025 Feb 17;13(2):195. doi: 10.3390/vaccines13020195 (PMC11861348; doi:10.3390/vaccines13020195)

Figure S3. Effect of AmoG on gut microbiota *in vivo*. Crucian carps were infected with *Aeromonas hydrophila* CCL1, Δ*AmoG*, Δ*AmoG*-C or PBS (control) for 24 h. (**A** and **B**) Alpha-diversity of microbiota in the distal gut was determined by QIIME (V1.9.1). (**C**) Bray-Curtis dissimilarity in the distal gut microbial community was determined by principal coordinates analysis (PCoA). (**D**) The average relative abundance of microbiota at the phylum level. (**E**) Differential microbial abundance shown by LDA SCORE. Mann-whiteney test.

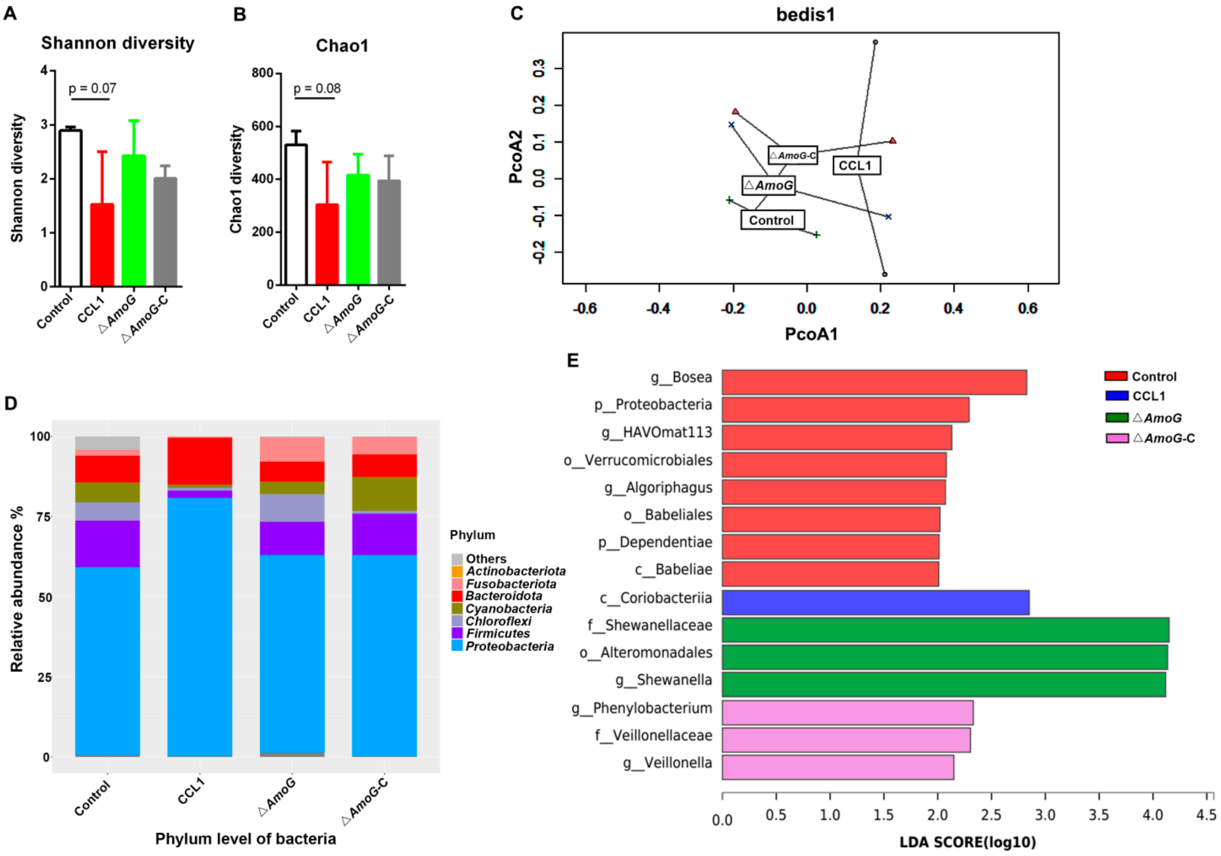

Supplement: Supplementary file 1 [file vaccines-13-00195-s001.zip › Figure S3.pdf]
