# Supplementary material for: Amonabactin Synthetase G Regulates Aeromonas hydrophila Pathogenicity Through Modulation of Host Wnt/β-catenin Signaling
Source: Vaccines (Basel). 2025 Feb 17;13(2):195. doi: 10.3390/vaccines13020195 (PMC11861348; doi:10.3390/vaccines13020195)

Figure S4. Summary depiction suggesting a possible mechanism by which *Aeromonas hydrophila* AmoG modulates the infectivity through the manipulation of the Wnt/β-catenin pathway.

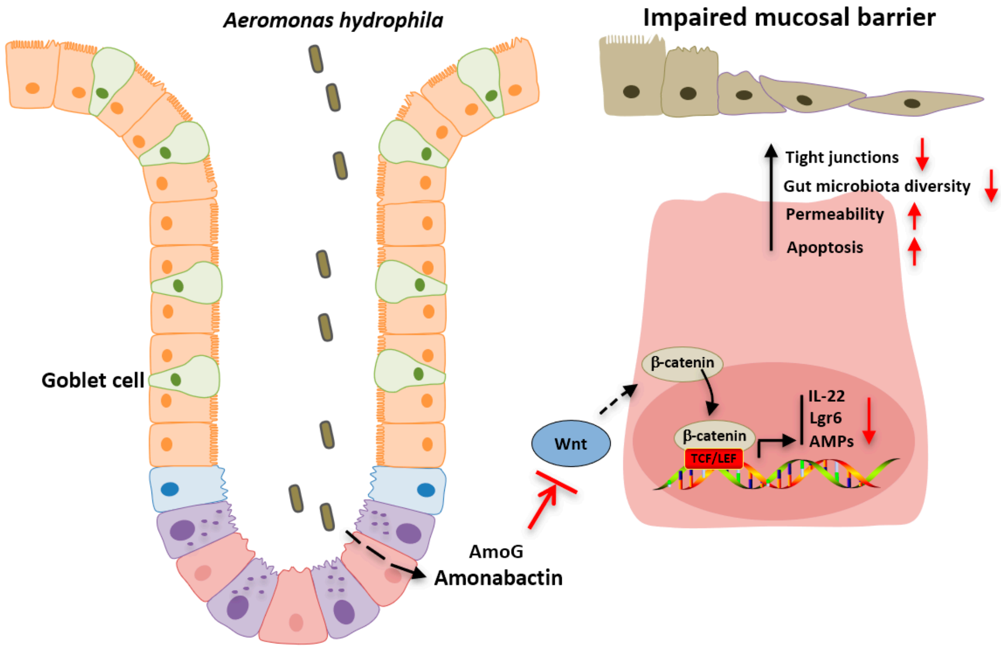

Supplement: Supplementary file 1 [file vaccines-13-00195-s001.zip › Figure S4.pdf]
