# Supplementary material for: Amonabactin Synthetase G Regulates Aeromonas hydrophila Pathogenicity Through Modulation of Host Wnt/β-catenin Signaling
Source: Vaccines (Basel). 2025 Feb 17;13(2):195. doi: 10.3390/vaccines13020195 (PMC11861348; doi:10.3390/vaccines13020195)
Supplement: Supplementary file 1 [file vaccines-13-00195-s001.zip › Table S1.pdf]

Table S1. PCR primers used in this study.

Primers	Sequence (5'-3')	PCR	Gene	Accession no.
AmoG-F1	ggatccCGCCTGCTGCACCTGATGCGC (BamHI)	Mutant construction	*AmoG*	CP092356
AmoG-R1	CATATGGCTGGCACCCAGCGCAAGCTGGTGCAGGTC			
AmoG-F2	GGTGCCAGCCATATGATTTTGCCGACC	Mutant construction	*AmoG*	CP092356
AmoG-R2	ggatccCCAGCAGAGGCGCGCCGGCAC (BamHI)			
AmoG-F3	gatatcATCGAGCGGCGCGCTATTCAGTTG (EcoRV)	Mutant construction	*AmoG*	CP092356
AmoG-R3	gatatcGCGCAGCTTGATCTGCTCGTCTAT (EcoRV)			
β-actin-F	TGGACTTTGAGCAGGAGATGG	qRT-PCR	*β-actin*	AF079831.1
β-actin-R	CTAGGAAGGATGGCTGGAAAA			
wnt3a-F	CAGCCTGACTTTCGGGTGAT	qRT-PCR	*wnt3a*	NC_039269.1
wnt3a-R	GTTTCGGTAGGGGGCTTGAA			
wnt10b-F	AGACTTTGCAGAGGAGTGGC	qRT-PCR	*wnt10b*	NC_039290.1
wnt10b-R	AACCGTACACCAAGACGGAC			
ctnnb1-F	GAGGAATCTCTCAGACGCCG	qRT-PCR	*ctnnb1*	XM_059567370.1
ctnnb1-R	GGTTGGACAGGATTCCAGCA			
Lgr6-F	AGCTTCAAGAGTTCCCCGTG	qRT-PCR	*Lgr6*	XM_026199275.1
Lgr6-R	GGTTCCCCACAAACGCTTTC			
ZO-1-F	GTTTGCCGTTGTAAAGGGTGT	qRT-PCR	*ZO-1*	HQ656016.1
ZO-1-R	TGGCTCAGAGGAAGAAAGATG			
Occludin-F	GGACGACTGGGCCATTGGGTA	qRT-PCR	*Occludin*	HQ110086.1
Occludin-R	CAGGCAGGGCAAGGGTTTCAT			
Claudin1-F	TTGAGTTCGGGAAGGCTCTG	qRT-PCR	*Claudin-1*	XP_026062913.1
Claudin1-R	GCGGTCGGTTCCAGGTTTTC			
Claudin2-F	ACACTTCTCTCCTTCATCAGAC	qRT-PCR	*Claudin-2*	XM_026198949.1
Claudin2-R	CTCCATCCTTCGACTTCTTGCT			
Claudin4-F	GCTTTCTTATTCGCTGGCGG	qRT-PCR	*Claudin-4*	XP_026134569.1
Claudin4-R	TGATAAGCCATGGGTGCGTT			
Claudin8-F	ATAGACTTTGCATTGCATTCG	qRT-PCR	*Claudin-8*	HQ656014.1
Claudin8-R	ATCTATGTGCTGTGAGGTGGTT			
MUC2-F	TCAGCAGGGTGTCCATTTCC	qRT-PCR	*MUC2*	XP_026057609.1
MUC2-R	CACAAGGATCCCTGCGACAT			
Hepcidin-1-F	CGTTCCCTTCACACAGCAGAC	qRT-PCR	*Hepcidin-1*	XP_026083628.1
Hepcidin-1-R	TACCCACAGCCTTTGTTGCG			
LEAP-2-F	GCGCTCGTTACTCTGGAGATG	qRT-PCR	*LEAP-2*	XP_026052780.1
LEAP-2-R	TCACGCCTGTTGAGCTTTATTG			
IL-22-F	GAAGATCTGCTGCCTCCACGCCA	qRT-PCR	*IL-22*	MR343566
IL-22-R	GCAGAAGTCCTGCAGGTACGTG			
HlyA-F	TACCTCAACGTCAACCGCAA	qRT-PCR	*HlyA*	XIH95476.1
HlyA-R	CCGGTAAACCAGCGAGATGT			
